# Linking People and Place: Facilitators & Barriers to Implementing Lifestyle Redesign in French-Canadian Context

**DOI:** 10.1093/geroni/igaf122.1716

**Published:** 2025-12-31

**Authors:** Marie-Hélène Lévesque, Mélanie Levasseur

**Affiliations:** Université de Sherbrooke, Sherbrooke, Quebec, Canada; Université de Sherbrooke, Sherbrooke, Quebec, Canada

## Abstract

Lifestyle Redesign is a well-recognized group-based occupational therapy approach to foster healthy lifestyles and reduce social isolation and loneliness among older adults. Mainly studied for its effects and cost-effectiveness, little is known about what influences its implementation. Drawing on insights from occupational therapists (OTs), OT students and expert-partners, this pre-implementation study explored facilitators and barriers to implementing the French-Canadian version (Remodeler sa vie®). Guided by the Consolidated Framework for Implementation Research, an action research involved 58 purposefully recruited participants, including experienced OTs from public and private sectors, OT students in entrepreneurial stream, and expert-partners with diverse backgrounds (e.g. gerontology, decision-making, and community services). Eleven focus groups were conducted using two semi-structured interview guides led by two OTs trained in qualitative methods. Participants (86.2% women, aged 20 to 72; mean = 43.2, SD = 12.5) identified 36 influential factors, with public-sector OTs encountering the most barriers to implementation (17) compared to those in private practice (12) and expert-partners (7). Key enablers were the approach’s strong evidence, relevance to aging populations, engagement of key actors, and growing interest in health self-management. Barriers stemmed from approach complexity (e.g., duration), resource constraints (e.g. human), socio-political challenges, implementation demands, and uncertainty around older adults’ engagement. Equitable access was a common concern, notably regarding socioeconomic status, geographical location and disability profiles. Findings supported a detailed implementation plan with 7 key axes, 20 objectives, and 34 actions to help Remodeler sa vie reach its full potential in promoting healthy lifestyles and reducing social isolation and loneliness.

